# Ishophloroglucin A Ameliorates VEGF-Induced Epithelial-Mesenchymal Transition via VEGFR2 Pathway Inhibition in Microgravity-Stimulated Human Retinal Pigment Epithelial Cells

**DOI:** 10.3390/antiox11112212

**Published:** 2022-11-08

**Authors:** Myeongjoo Son, Bomi Ryu, Jun-Geon Je, You-Jin Jeon, Dae Yu Kim

**Affiliations:** 1 Inha Research Institute for Aerospace Medicine, Inha University, Incheon 22212, Korea; 2 Major of Food Science and Nutrition, Pukyong National University, Busan 48513, Korea; 3 Department of Marine Life Sciences, Jeju National University, Jeju 63243, Korea; 4 Marine Science Institute, Jeju National University, Jeju 63333, Korea; 5 Center for Sensor Systems, Inha University, Incheon 22212, Korea; 6 Department of Electrical Engineering, College of Engineering, Inha University, Incheon 22212, Korea

**Keywords:** retinal pigment epithelium, epithelial-mesenchymal transition, microgravity, Ishige okamurae, ishophloroglucin A, vascular endothelial growth factor, vascular endothelial growth factor receptor 2 signaling

## Abstract

Microgravity stimulation is associated with retinal pigment epithelial (RPE) cells that transition to mesenchymal cells (EMT), and these pathological changes cause visual impairment. Vascular endothelial growth factor (VEGF) is produced from the RPE and contributes to photoreceptor survival. However, changes in VEGF production and function under microgravity stimulation are unknown. In this study, we verified that microgravity stimulation changed the morphological characteristics of human RPE cells (ARPE19 cells) and the expression of actin cytoskeleton regulators, which are related to excessive VEGF expression. Interestingly, microgravity stimulation increased not only the production of VEGF but also the expression of EMT markers. Previously, we studied the potential of ishophloroglucin A (IPA), a phlorotannin, as an antioxidant. In silico results confirmed that IPA could structurally bind to VEGF receptor 2 (VEGFR2) among VEGFRs and inhibit the VEGF pathway. IPA significantly decreased VEGF production and EMT marker expression in microgravity-stimulated cells. It also significantly reduced excessive cell migration in VEGF-induced EMT. Overall, our findings suggested that IPA treatment decreased VEGF production and EMT marker expression in microgravity-stimulated or VEGF-treated ARPE19 cells, and this decrease in EMT could restore excessive cell migration by inhibiting the VEGF/VEGFR2 pathway. Therefore, it is a potential therapeutic candidate for angiogenesis-related eye diseases.

## 1. Introduction

Humans exposed to microgravity have exhibited multisystem changes, including bone demineralization [1], muscle atrophy [2], immune system dysregulation [3], cardiovascular dysfunction [4], and difficulties with vestibular processing [5]. Functional and structural changes in the ocular system, consisting of the eye and its central visual system, have also been reported [6]. These health problems are associated with oxidative stress. “Space flight-associated neuro-ocular syndrome” is characterized by microgravity-exposed ocular features such as optic disc edema, globe flattening, hyperopic refractive error shifts, choroidal and retinal folds, and focal areas of the ischemic retina [6]. These features are related to visual impairment, which is considerably associated with the retina, a light-sensitive layer of the eye. The retinal pigment epithelium (RPE) is formed from modified neuroepithelial cells and is a single layer of pigment cells with lipofuscin and melanin. It plays an essential role in visual function [7].

Numerous microvilli surround the outer segment of photoreceptors, thereby increasing the surface area in contact with the outer segment of photoreceptors by approximately 30-fold. They promote increased cell contact and accommodation [8]. The RPE forms the outer blood–retina barrier to the systemic circulation [9]. This barrier regulates and filters the molecular movement of nutrients and solutes from the choroid to the subretinal space. When this barrier is disrupted, blood-derived proteins and other potentially toxic solutes accumulate within the retina [10]. The RPE not only supports peripheral cells and forms a barrier but also abundantly expresses and secretes proteins, including vascular endothelial growth factor (VEGF) and pigment epithelium-derived factor (PEDF). VEGF is normally secreted in the basal direction to promote choroidal vessel growth, but excessive preexisting vessel growth underlies major blinding retinal diseases [11].

The disruption of the blood–retina barrier contributes to the pathogenesis of various cases of retinopathy. The RPE–mesenchymal transition (EMT) plays a key role in retinopathies such as age-related macular degeneration (AMD), the leading cause of severe and permanent visual impairment. However, EMT can be activated under pathological conditions, such as wound healing, inflammation, and oxidative stress. Traditionally, EMT is defined as a cellular and molecular process [12] by which cells lose their epithelial properties; it is characterized by apical–basal polarity and stable intercellular junctions. It also acquires a mesenchymal phenotype, including cytoskeletal and morphological rearrangements. Thus, they obtain fibroblast-like gene expression profiles, migratory abilities, and the ability to generate an extracellular matrix (ECM) [13,14]. In the first step of EMT, core proteins such as E-cadherin, claudins, and occludin that maintain lateral contact between adjacent epithelial cells through adherent junctions are downregulated [15]. In the next step, interactions with the ECM matrix and polarity decrease, and cytoskeletal reorganization and cell migration increase. Snail, a key transcription factor, is a zinc-finger transcriptional repressor that controls EMT during embryogenesis and tumor progression [16]. In the last step, the expression levels of vimentin, N-cadherin, and α-smooth muscle actin (α-SMA), which are mesenchymal markers, increase. The cellular-to-mesenchymal transition is accompanied by ECM remodeling proteins (matrix metalloproteinases, MMPs, and TIMPs) and ECM deposition (collagens and fibronectin) [17]. Various characteristics of the EMT response have been observed in the RPE of patients with dry AMD [18].

As is well known, VEGF receptors (VEGFRs) have three main subtypes: VEGFR1, VEGFR2, and VEGFR3; all VEGF family members bind to VEGFRs on the cell surface and stimulate cellular responses, leading to dimerization and activation through transphosphorylation [19]. Among them, VEGF-VEGFR2 (also known as the kinase insert domain-containing receptor, KDR, or fetal liver kinase-1, Flk-1) [20] binding in cancer cells stimulates the secretion of MMPs, leading to ECM degradation and providing a pathway for cells to invade nearby tissues. VEGF-VEGFR2 binding promotes tumor progression and migration [21,22,23] and can regulate Snail and β-catenin proteins through a mitogen-activated protein kinase (MAPK) cascade. These molecules are activated for EMT and cell invasion in breast cancer [24]. Further studies on the relationship between microgravity stimulation, VEGF signaling, and EMT in the eye are essential.

Ishophloroglucin A (IPA) is a phlorotannin derived from brown algae (Ishige okamurae), which is abundantly distributed in the coastal areas of the temperate coastal zone of East Asia [25]. Previously, we showed that IPA has a potential preventive effect on the angiogenesis of vascular endothelial cells in mimicked diabetic conditions [26]. However, the previous study did not demonstrate whether IPA can directly inhibit VEGF signaling [26]. In this study, we observed that microgravity stimulation increased VEGF expression and induced EMT in human retinal pigment epithelial cells. Furthermore, we demonstrated that IPA could inhibit microgravity-stimulated or VEGF-induced EMT by reducing VEGF-VEGFR2 signaling.

## 2. Materials and Methods

### 2.1. IPA Preparation and In Silico Binding Analysis of IPA-VEGFRs

IPA (98% purity; Aktin Chemical Inc., Chengdu, China) identified in Ishige okamurae was subjected to a docking study to explore its binding to the VEGFR enzyme. All modeling experiments were performed using the CDOCKER INTERACTION program (Discovery Studio 2018, Accelrys, Inc., San Diego, CA, USA), which provides a unique set of tools to model protein–ligand interactions and predicts how a small flexible molecule such as a substrate or a drug candidate binds to a 3D protein structure represented by grid interaction potentials. In each experiment, the biological target VEGFRs downloaded from the Protein Data Bank (http://www.pdb.org, accessed on 1 May 2022, VEGFR1 [ID: 5ABD] and VEGFR2 [ID: 2OH4]) were used. The homology model of VEGFR3 was used as described by Li [27]. The protein-ligand complex, binding potential, the precise location of the binding site, charges, and the addition of hydrogen atoms were assessed using the flexible docking program.

### 2.2. Experimental Cell Model

#### 2.2.1. ARPE19 Cell Culture

Immersive human retinal pigment epithelial cells (ARPE19) were used in this study in order to demonstrate microgravity-stimulated RPE models. ARPE19 cells are frequently used to research the retina and have become a good alternative model. However, ARPE19 cells have a functional passage limitation (~p20), and high-passage cells have some differentiating characteristics, for example, pigmentation [28].

ARPE19 cells were purchased from ATCC (VA, USA), and the cells were cultured in Dulbecco’s Modified Eagle’s Medium/F12 (Welgene, Daegu, Korea) containing 10% fetal bovine serum (FBS; Welgene) and 100 U/mL penicillin–streptomycin (Welgene) in a CO_2_ incubator at 37 °C. Cells in passages 5–10 were used in all experiments.

#### 2.2.2. Clinostat Setup

A gravity controller was used for 3D clinostat exposures (Gravite ^®^, Space Bio-Laboratories Co., Ltd., Japan) [29]. ARPE19 cells were seeded in 25 cm^2^ center-sealed tissue culture flasks (BD Falcon, NJ, USA) and properly attached to the bottom of the flask for at least 24 h before the flask was filled with a growth medium. This device generates microgravity similar to space gravity. Microgravity (10^−3^ × g) is generated by rotating a sample around two axes and integrating the gravity vector and the time axis. Within 8 min, these specific conditions favor the formation of an environment of 10^−3^ × g, as measured by a gravitational accelerometer. This environment is defined to simulate microgravity [30,31,32]. In the present study, ARPE19 cells were mainly separated into two groups that were exposed to either normal gravity (1G group, without rotation) or simulated microgravity (SMG group, clinorotation at 30 rpm) for 1, 3, 5, and 7 days and incubated at 37 ℃ and 5% CO2. An SMG/IPA group was prepared by treating the SMG group with IPA for 7 days. All cells and conditioned media were transferred to new tubes and stored in a deep freezer.

### 2.3. RNA Extraction and cDNA Synthesis

ARPE19 cells were mixed with 1 mL of TRIzol (Invitrogen, Waltham, MA, USA). Homogenates were added to 0.2 mL of chloroform (Invitrogen), thoroughly mixed, and centrifuged at 14,000 × *g* and 4 °C for 15 min. Aqueous phases were transferred to new tubes, mixed with 0.5 mL of isopropanol (Invitrogen), and centrifuged under the same conditions. The isolated total RNA was washed with 70% ethanol and dissolved in 10–30 µL of nuclease-free water.

### 2.4. Quantitative Real-Time Polymerase Chain Reaction (qRT-PCR)

qRT-PCR was performed to verify the gene expression levels. Primers (Table S1), 800 ng of template DNA, and 2X QGreenBlue qPCR Master Mix (CellSafe, Seoul, Korea) were mixed gently and placed in a thermal cycler (Bio-Rad, Hercules, CA, USA). The following protocol was performed: pre-denaturation at 95 °C for 3 min, denaturation at 95 °C for 10 s, annealing at 58 °C for 10 s, and read extension and fluorescence at 72 °C for 10 s. In the last step, the melting curve was analyzed.

### 2.5. Protein Extraction

The cells were scraped with RIPA lysis buffer (ATTO; Tokyo, Japan) containing protease and phosphatase inhibitors to isolate proteins from ARPE19 cells. After centrifugation at 13,000× *g* and 4 °C for 20 min, the supernatant was collected in a new tube and analyzed for protein concentration by using a Pierce BCA protein assay kit (Thermo Fisher Scientific, Inc., Waltham, MA, USA). Western blotting was performed to verify protein expression.

### 2.6. Western Blotting

In this procedure, 20 μg of total protein per lane was separated through 10% hand-made SDS-PAGE gel electrophoresis to estimate the protein expression in ARPE19 cells. The proteins were transferred to polyvinylidene fluoride (PVDF) membranes at 500 mA for 20 min by using semi-dry blotting (ATTO). Then, 5% skim milk (*v/v*) was used as a blocking solution to decrease non-specific binding. The PVDF membranes were immersed with primary antibodies (Table S2) at 4 °C for 2 days, washed with tris-buffered saline with 0.1% Tween 20 (TTBS) thrice, and immersed with horseradish peroxidase (HRP)-conjugated secondary antibodies at room temperature for 1 h. ARPE19 cell protein extracts were obtained using RIPA lysis buffer (ATTO) containing a protease and phosphatase inhibitor cocktail (ATTO). Western blotting signals were detected using the ChemiDoc XRS+ system and quantified using Image J (NIH, Bethesda, MD, USA).

### 2.7. Indirect Enzyme-Linked Immunosorbent Assay (ELISA)

Transforming growth factor beta 2 (TGFβ2) and connective tissue growth factor (CTGF) were measured in the conditioned medium (CM), and VEGF was measured in the CM and cell lysate via indirect ELISA. Proteins from the conditioned medium and cell lysate were quantified using the BCA protein assay kit (Thermo Fisher Scientific, Inc.). A plate-coating solution mixture (0.6% sodium bicarbonate and 0.3% sodium carbonate in distilled water, pH 6.0) was incubated in a 96-well plate at 4 °C overnight. A blocking solution (5% skim milk containing 0.1% Triton X-100 in phosphate-buffered saline; TPBS) was incubated at 4 °C overnight. Unbound proteins were removed by washing with TPBS and then incubated with primary antibodies at room temperature for 6 h (Table S2). Unbound antibodies were removed by washing with TPBS and incubated with HRP-conjugated secondary antibodies at room temperature for 2 h. After the unbound antibodies were washed, the color was developed by incubating the sample with 3,3′,5,5′-tetramethylbenzidine for 15 min. Then, a 2 M sulfuric acid reaction stop solution was added to each well, and absorbance was measured at 450 nm by using an Epoch microplate spectrophotometer (BioTek Instruments Inc., Winooski, VT, USA).

### 2.8. Scratch-Wound Migration Assay and Transwell Migration Assay

A scratch-wound migration assay and a transwell migration assay were performed to examine the role of VEGF165 in ARPE19 cell migration. The ARPE19 cells were seeded at 4 × 10^4^ cells/mL in a 24-well plate and then treated with a starvation medium (FBS-free medium) for 24 h. The cells were treated with PBS (PBS group), 100 ng/mL recombinant human VEGF165 protein (Peprotech; NJ, USA, VEGF group), or VEGF plus 2.5 nM IPA (VEGF/IPA group) for 24 h and scratched with a 1 mL pipette tip to assess cell migration. The ability of cells to migrate and close the wound space was assessed 26 h after the scratch.

A transwell migration assay was conducted to measure the chemotactic capacity of VEGF. The ARPE19 cells were seeded in the upper chamber of a 24-well transwell plate (SPL Life Sciences, Pochon, Korea) for 12 h, and the growth medium was changed to a starvation medium for 24 h. In the bottom chamber, a new starvation medium with PBS (PBS group), 100 ng/mL VEGF (VEGF group), or VEGF plus 2.5 nM IPA (VEGF/IPA group) was placed for 30 h. Migrating cells passed through a membrane with a bottom pore size of 8 µm. Nonmigrating cells on top of the chamber were removed using cotton swabs, and migrating cells were fixed with 1% paraformaldehyde. Next, the cells were stained with hematoxylin for 1 min and quantified. The average wound space and migrating cells were measured using Image J software (NIH) under a light microscope.

### 2.9. Statistical Analysis

The Kruskal–Wallis test was used as a non-parametric method, and Mann–Whitney U was used as a post hoc test to verify significant differences among the groups. Results were expressed as mean ± standard error of the mean (SEM), and *p* < 0.05 indicated statistical significance. Means denoted by different letters indicate significant differences between groups (*p* < 0.05). Each experiment was performed three times. Statistical analysis was performed in SPSS version 22 (IBM Corporation, Westchester, NY, USA), and *p*-values are illustrated for each legend.

## 3. Results

### 3.1. Actin Cytoskeleton and Cell Population Changed during Microgravity-Stimulated Development in ARPE19 Cells

**Figure 1:**
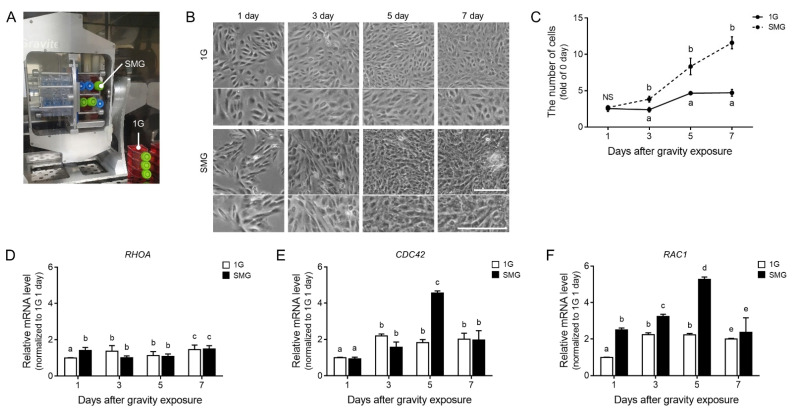
Increased cell proliferation and activated actin cytoskeleton regulator expression in microgravity-stimulated ARPE19 cells. (**A**) ARPE19 cells were grown in 25 cm^2^ flasks in the same incubator for 7 days. (**B**) Representative cell images show morphological changes following microgravity stimulation, and the changes can easily be found in enlarged images (bottom row). (**C**) The graph shows the quantified cell number of 1G- (white) or microgravity-stimulated ARPE19 cells (SMG, black). (**D**–**F**) Relative mRNA levels of (**D**) *RHOA*, (**E**) *CDC42*, and (**F**) *RAC1* were confirmed by qRT-PCR. Means denoted by different letters indicate significant differences between groups (*p* < 0.05). Scale bar = 200 µm; CDC42; cell division cycle 42, NS; not significant, RAC1; Rac family small GTPase 1, RHOA; Ras homolog family member A, SMG; simulated microgravity.

To establish a microgravity stimulation model in ARPE19 cells, we prepared two conditions. The simulated microgravity group (SMG) was exposed in a CO_2_ cell culture incubator and a clinostat, and the control group (1G) was only kept in the same CO_2_ cell culture incubator (Figure 1A). The cell shape and proliferation validated the changes (Figure 1B,C). After the microgravity stimulation, the number of apoptotic ARPE19 cells was not increased compared to 1G (Figure S1). Morphological changes in ARPE19 cells following stimulation verified that the number of ARPE19 cells increased. Their cell morphology became irregular and shrunken (Figure 1B,C).

Microgravity stimulation readily induces changes in the actin cytoskeleton by controlling the expression of actin and actin-related factors [32,33,34]. Ras homolog family member A (RhoA), cell division cycle 42 (CDC42), and Rac family small GTPase 1 (Rac 1) are key regulators of the actin cytoskeleton and can change with microgravity stimulation. As we expected, the mRNA expression of three genes (*RHOA*, *CDC42*, and *RAC1*) was changed. The mRNA expression levels of *CDC42* and *RAC1* were significantly the highest 5 days after microgravity stimulation, whereas the mRNA expression of *RHOA* was rarely changed after microgravity stimulation (Figure 1D–F).

### 3.2. VEGF Expression and EMT-Related Markers Increased during Microgravity-Stimulated Development in ARPE19 Cells

**Figure 2:**
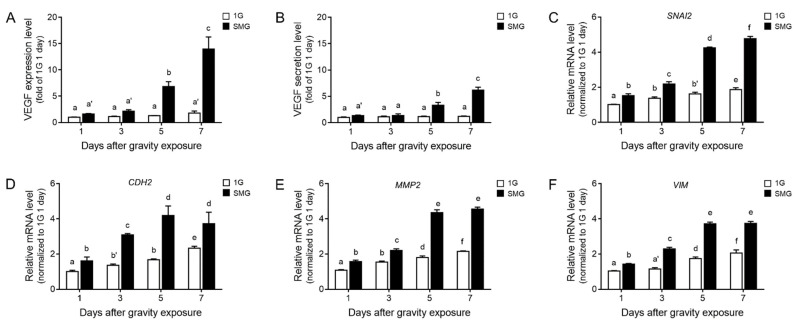
Increased VEGF level and EMT marker expression in microgravity-stimulated ARPE19 cells. (**A**) VEGF expression level in cell lysate and (**B**) secretion level in conditioned medium were validated by ELISA on days one, three, five, and seven of 1G (white) or microgravity stimulation (SMG, black). (**C**–**F**) Relative mRNA levels of (**C**) *SNAI2*, (**D**) *CDH2*, (**E**) *MMP2,* and (**F**) *VIM* were confirmed by qRT-PCR. Means denoted by different letters indicate significant differences between groups (*p* < 0.05). CDH2, cadherin 2; MMP2, matrix metalloproteinase 2; SNAI2, Snail family transcriptional repressor 2; SMG, simulated microgravity; VEGF, vascular endothelial growth factor; VIM, vimentin.

To validate the VEGF protein level in microgravity-stimulated ARPE19 cells, we assessed the VEGF expression and secretion levels from the cell lysate and conditioned medium via ELISA. With the extension of the microgravity stimulation time, the VEGF expression and secretion levels increased significantly 5 days after microgravity stimulation. The levels were the highest after 7 days (Figure 2A,B). In addition, changes in the mRNA of actin cytoskeleton regulators were the highest on day 5, while VEGF expression and secretion levels were the highest on day 7 after microgravity exposure.

To confirm the effect of excessively expressed and secreted VEGF on RPE cells, we validated the changes in EMT markers in microgravity-stimulated ARPE19 cells. The gene expression level of Snail2 (encoded by *SNAI2*), a key transitory epithelial–mesenchymal cell phenotype marker, and N-cadherin (cadherin 2, encoded by *CDH2*) increased significantly 5 days after microgravity stimulation. The highest levels were observed after 7 days (Figure 2C,D). The expression levels of MMP2 and vimentin (encoded by *VIM*) also increased significantly 5 days after microgravity stimulation, and the highest levels were obtained after 7 days (Figure 2E,F). Interestingly, simulated microgravity increased VEGF expression; similarly, it significantly increased the secretion and expression levels of EMT markers from day 5 (Figure 2).

### 3.3. IPA Disrupted VEGFR2 Signaling Activation by Inhibiting VEGF–VEGFR2 Binding in Microgravity-Stimulated ARPE19 Cells

**Figure 3:**
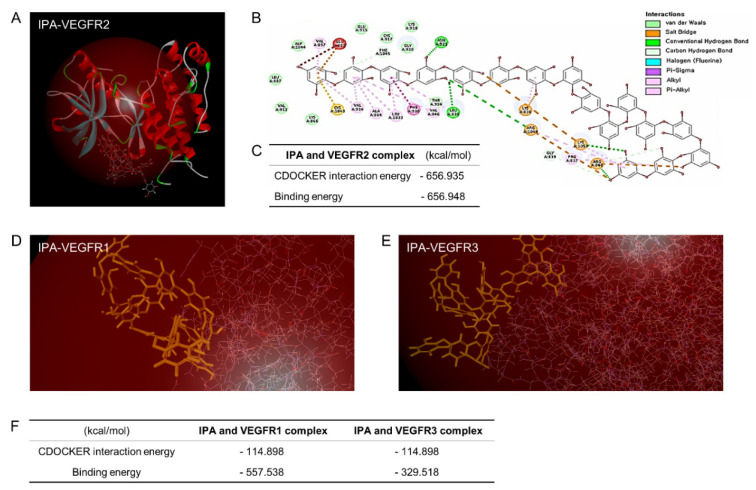
Computational docking prediction of IPA with VEGFRs. (**A**) Three- and (**B**) two-dimensional diagrams of the IPA-VEGFR2 complex were computationally predicted by in silico methods, and (**C**) the table shows IPA-VEGFR2 binding energy and interaction energy. In addition, the computational prediction shows a 3D simulation of (**D**) IPA-VEGFR1 and (**E**) -VEGFR3 docking. (**F**) The table indicates the results of the binding energy and CDOCKER interaction energy of the docking of IPA-VEGFR1 and IPA-VEGFR3.

We performed an in silico study of molecular docking to obtain insights into the binding modes of VEGFRs and IPA. The crystal structures of VEGFRs were obtained from the Protein Data Bank. The computational predictions of a VEGFR structure are provided in Figure 3. Among the three types of VEGFRs, VEGFR2 (−656.948 kcal/mol) had stronger binding energy with IPA than IPA-VEGFR1 (−557.538 kcal/mol) and IPA-VEGFR3 (−329.518 kcal/mol). The CDOCKER energy values were −656.935 kcal/mol in IPA-VEGFR2 and −114.898 kcal/mol in IPA-VEGFR1 and IPA-VEGFR3 (Figure 3D–F). This computational docking prediction showed that IPA powerfully inhibited VEGF-VEGFR2 binding compared to VEGFR1 or VEGFR3. We also performed a computational prediction of the benzimidazole urea–VEGFR2 binding energy.

Benzimidazole urea is a VEGFR2 antagonist chemical [35], whose binding inactivates the receptor and inhibits angiogenesis. Interestingly, the benzimidazole urea–VEGFR2 binding energy was lower than that of IPA-VEGFR2 (Figure S2). The examination of the best docking mode revealed an electrostatic interaction and a network of hydrogen bonds in the IPA and VEGFR2 complex.

**Figure 4:**
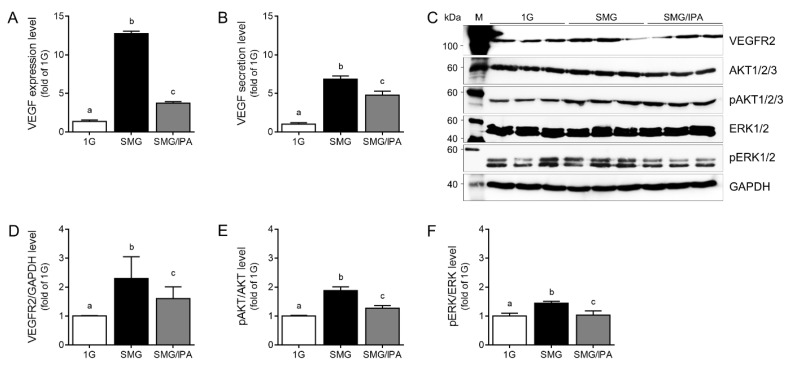
The inhibitory effects of VEGFR2 signaling by VEGFR2 docking with IPA in microgravity-stimulated ARPE19 cells. (**A**) VEGF expression levels in cell lysate and (**B**) secretion levels in conditioned medium were detected by ELISA on day 7 after microgravity stimulation with or without IPA. (**C**) Expression levels of VEGFR2 and its related proteins, including AKT and ERK, in microgravity-stimulated ARPE19 cells with PBS (SMG; black) or 2.5 nM IPA (SMG/IPA; gray) were validated by Western blotting. (**D**) VEGFR2, (**E**) phosphorylated AKT, and (**F**) ERK were quantified by Image J software. Means denoted by different letters indicate significant differences between the groups (*p* < 0.05). SMG, simulated microgravity; IPA, ishophloroglucin A; VEGF, vascular endothelial growth factor; VEGFR2, vascular endothelial growth factor receptor 2.

VEGF secretion by the RPE is due to the activation of signaling pathways involving VEGF–specific tyrosine kinase receptors, whose activation loops are generated by inducing a phosphorylation cascade [36]. Autocrine VEGF-VEGFR2 signaling induces an intracytoplasmic signaling cascade that phosphorylates the rat sarcoma virus (Ras)/rapidly accelerated fibrosarcoma (Raf)/extracellular regulated kinase 1/2 (ERK 1/2) and phosphoinositide 3-kinase (PI3K)/AKT proteins [36,37]. The VEGF expression in cell lysate and secretion in the conditioned medium were higher in the microgravity-stimulated ARPE19 cell group after 7 days (SMG) than in 1G as the control. The values in the IPA-treated SMG group (SMG/IPA) were lower than in SMG (Figure 4A,B). The VEGFR2 expression level was higher in SMG than in 1G. IPA decreased the VEGFR2 expression more than SMG did (Figure 4C,D). The level of phosphorylated AKT (Thr 308 and Ser 473) was higher in SMG than in 1G, but it was lower in SMG/IPA than in SMG (Figure 4C,E). IPA treatment also affected ERK1/2 (Thr202 and Tyr204) phosphorylation in the microgravity-stimulated ARPE19 cell group (Figure 4C,F).

### 3.4. IPA Protected Microgravity-Induced EMT in ARPE19 Cells

**Figure 5:**
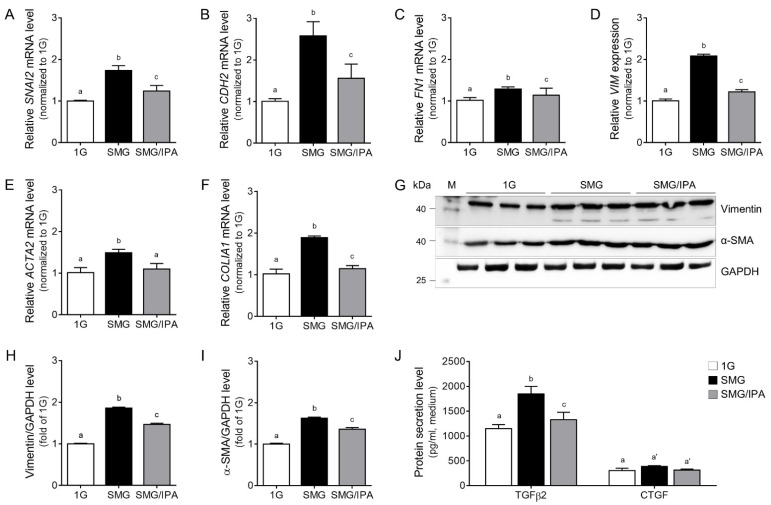
The inhibitory effects of the EMT process in IPA-treated microgravity-stimulated ARPE19 cells. (**A**–**F**) EMT-related factor mRNA levels in 1G- (white), SMG- (black), or SMG/IPA-treated (gray) ARPE19 cells were measured by qRT-PCR analysis. (**G**–**I**) Vimentin (encoded by VIM) and α-SMA (encoded by ACTA2) protein expression levels in 1G- (white), SMG- (black), or SMG/IPA-treated (gray) ARPE19 cells were measured by Western blot analysis. (**J**) Secreted proteins, TGFβ2 and CTGF, in the ARPE19 cell culture medium were measured by ELISA. Means denoted by different letters indicate significant differences between the groups (*p* < 0.05). ACTA2, Actin Alpha 2, Smooth Muscle; CDH2, cadherin 2; COLIA1, collagen type 1 alpha 1; CTGF, connective tissue growth factor; FN1, fibronectin 1; GAPDH, glyceraldehyde 3-phosphate dehydrogenase; IPA, ishophloroglucin A; SMG, simulated microgravity; SNAI2, Snail family transcriptional repressor 2; TGFβ2; transforming growth factor beta 2; VIM, vimentin.

We validated EMT markers, including Snail 2, N-cadherin, FN1 gene coding protein (fibronectin 1; encoded by *FN1*), vimentin, α-SMA (encoded by *ACTA2*), and collagen 1 (encoded by *COL1A1*), in SMG. IPA reduced the expression of EMT markers compared to SMG (Figure 5A–F). Among them, vimentin and α-SMA protein expression, as representative EMT markers, were validated in SMG. Furthermore, IPA reduced the expression of EMT markers compared to SMG, as shown in Figure 5G–I.

Next, we checked transforming growth factor beta 2 (TGFβ2) and connective tissue growth factor (CTGF) in the ARPE19 cell culture medium. These two factors are representative of EMT regulators [38]. Especially the TGFβ2 secretion level increased in the conditioned medium, but there was no significant CTGF secretion in SMG. IPA treatment decreased the TGFβ2 secretion level from ARPE19 cells, as shown in Figure 5J.

### 3.5. IPA Reduced Abnormal Migration and EMT in VEGF-Treated ARPE19 Cells

**Figure 6:**
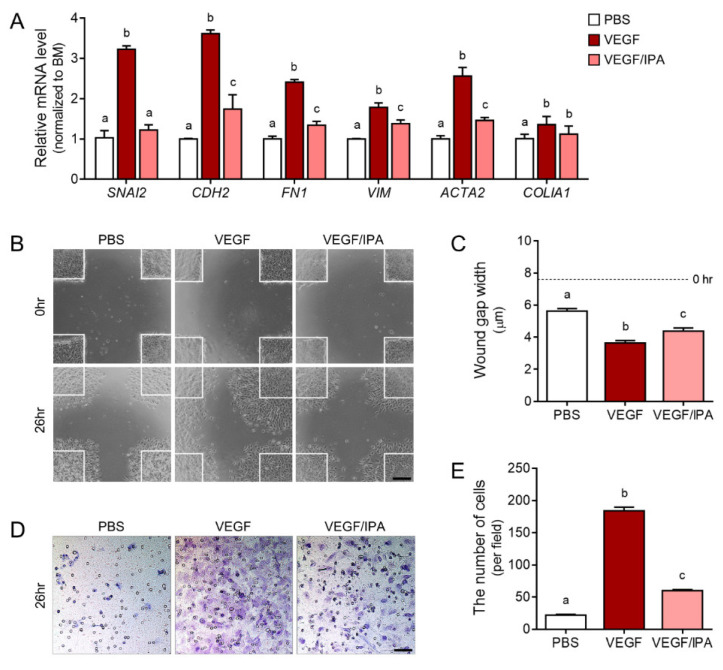
The inhibitory effects of the migratory mesenchymal phenotype on the EMT process in IPA-treated ARPE19 cells. (**A**) EMT-related factor mRNA levels in PBS- (white), VEGF- (red), or VEGF/IPA-treated (pink) ARPE19 cells measured by qRT-PCR analysis. (**B**) Representative bright-field images show that VEGF resulted in a significantly increased migration speed compared with PBS. This acceleration of gap closure was also prevented by IPA treatment. (**C**) Wound closure is expressed as the remaining area uncovered by the ARPE19 cells. The wound gap at time point 0 h is set in the quantified graph. (**D**) Representative images of the transwell migration assay of ARPE19 cells with PBS, VEGF, or VEGF/IPA. Hematoxylin-stained ARPE19 cells show a purple color, and (**E**) the graphical representation of the transwell migration assay of ARPE19 cells with VEGF/IPA shows a reduction in transwell migration compared to VEGF. Means denoted by different letters indicate significant differences between the groups (*p* < 0.05). Scale bar = 100 µm; ACTA2, Actin Alpha 2, Smooth Muscle; CDH2, cadherin 2; COLIA1, collagen type 1 alpha 1; FN1, fibronectin 1; IPA, ishophloroglucin A; SNAI2, Snail family transcriptional repressor 2; VEGF, vascular endothelial growth factor; VIM, vimentin.

We confirmed that VEGF expression and secretion by microgravity stimulation induced EMT in ARPE19 cells, but this process could be inhibited by IPA treatment. To directly determine the function of VEGF in the RPE, we treated the ARPE19 cells with PBS (PBS group), VEGF (VEGF group), and VEGF plus IPA (VEGF/IPA group). First, we validated EMT markers, including Snail 2, N-cadherin, fibronectin 1, vimentin, α-SMA, and collagen 1, via qRT-PCR. The gene expression levels of the EMT markers were higher in the VEGF group than in the PBS group, but the levels in the VEGF/IPA group were lower than those in the VEGF group (Figure 6A).

In addition to EMT gene expression changes due to excessive VEGF, epithelial cells acquire a migratory and invasive mesenchymal phenotype in EMT. The excessive migration and wound gap width of the VEGF-induced ARPE19 cells were lower than those in the VEGF group. The width in the VEGF/IPA group was higher than that in the VEGF group (Figure 6B,C). We measured the chemotactic capability of ARPE19 cells toward VEGF through the transwell cell migration assay. The number of migrating cells that crossed the bottom was higher in the VEGF group than in the PBS group, but the number of cells in the VEGF/IPA group was lower than that in the VEGF group (Figure 6D,E). IPA could function as a VEGF–VEGFR2 signaling inhibitor. Direct VEGF treatment and microgravity stimulation induced EMT, but IPA treatment reduced cell migration (Figure 6). Therefore, IPA can be a therapeutic VEGFR2 inhibitor candidate for various angiogenesis-related eye diseases.

## 4. Discussion

Cellular responses to mechanical stress have been well documented, but the responses that occur when cells are subjected to mechanical stress conditions are yet to be fully described. The most pronounced cellular changes after exposure to microgravity are variations in cell adhesion properties, shape, size, and volume [39,40,41]. Space environment exposure increases oxidative stress in the retina, and the retina thickness significantly decreases. Various compensatory mechanisms reduce cellular damage related to oxidative stress [42].

Microgravity-induced changes in cell morphology reflect changes in the cytoskeletal structure, namely, microtubules and actin filaments (F-actin), as cells sense a reduced gravitational load and mechanical unloading [43,44,45]. However, the actin cytoskeleton’s response to microgravity is poorly elucidated. Some studies have reported that exposure to microgravity reduces the expression of actin and actin-related proteins (i.e., actin-related protein 2/3 complex (Arp2/3) and RhoA), resulting in the disassembly of the actin cytoskeleton [32,33,34]; conversely, other studies have shown that increased F-actin and stress fiber formation are associated with the development of lamellipodium protrusions after exposure to microgravity [46].

We believe that microgravity stimulation affects the actin cytoskeleton. As we expected, the expression of *CDC42* and *RAC1* increased in microgravity-stimulated ARPE19 cells (Figure 1). Among actin cytoskeleton regulators, Cdc42 and Rac1 are associated with VEGF-mediated angiogenesis, including endothelial cell migration, invasion, and proliferation [47,48,49,50]. Therefore, microgravity stimulation can modulate actin cytoskeleton regulators and trigger VEGF expression and secretion in ARPE19 cells (Figure 1 and Figure 2A,B). Microgravity stimulation induced ARPE19 cells formed by partially multicellular spheroids. Corydon showed that the expression levels of VEGF and FLK1 differed from those of adherent cells according to the shape of microgravity-stimulated ARPE cells [43].

EMT is a process by which epithelial cells lose cell polarity and intercellular adhesion and acquire migratory and invasive properties to become mesenchymal stem cells, which are pluripotent stromal cells that can differentiate into various cell types. EMT is initiated and completed via different molecular processes, including the activation of transcription factors, the expression of specific cell surface proteins, the reorganization and expression of cytoskeletal proteins, and the production of ECM-degrading enzymes. Well-known biomarkers, including Snail, vimentin, fibronectin, and α-SMA, are used to detect cells passing through EMT [51]. In the present study, the expressed and secreted VEGF increased the EMT-related expression of genes, including *SNAI2*, *CDH2*, *VIM*, *FN1*, and *ACTA2*, in microgravity-stimulated ARPE19 cells via an autocrine pathway (Figure 2). However, studies have described VEGF overexpression that induces EMT, leading to cancer metastasis through an autocrine loop [19]. VEGF-overexpressing cancer cells show increased tumorigenicity, invasiveness, proliferation, and EMT characteristics, including mesenchymal marker expression (N-cadherin, Snail2, and vimentin). Moreover, VEGF remarkably changes the cell morphology and cellular transcriptome via autocrine mechanisms involving cytoskeleton-related signaling pathways [52].

EMT plays a key role in the pathogenesis of eye diseases, especially AMD, which leads to profound and permanent vision loss. RPE cells lose their cell–cell adhesion and apical–basal polarity to transform into mesenchymal cells under EMT [50]. In addition, direct VEGF-treated ARPE19 cells show increases in both EMT marker expression and excessive cell migration (Figure 6). Among various attempts to suppress EMT, anti-VEGF/VEGFR2 signaling is used to treat eye diseases. Intraocular injections of VEGF-neutralizing proteins, such as anti-VEGF therapy, are beneficial to patients with choroidal neovascularization. However, anti-VEGF injections increase the risk of RPE atrophy [53,54,55]. Despite these risks, VEGF signaling inhibitors are meaningful as a treatment for eye diseases. Molecular docking can predict the conformation of the specific potential binding mode of a ligand within the active site of a protein receptor with a known structure [56]. The interaction sites and energy values of VEGFRs, as modeled through in silico molecular docking simulations, have been explored in investigations into inhibitors to prevent or downregulate the signaling involved in angiogenesis-related eye diseases. As VEGFR inhibitors, several phenolic compounds or inhibitors with a metal-chelating ability have been widely studied in experimental and computational fields [57]. Our in silico results showed that IPA had the greatest binding energy with VEGFR2 among the VEGFRs, even benzimidazole urea, as a VEGFR2 antagonist (Figure 3A–C and Figure S2). IPA could function as a VEGF–VEGFR2 signaling inhibitor. In addition, IPA contains hydroxyl groups (−OH) bound to its benzene ring, and the high number of hydroxyl groups may benefit its pharmacological activities [58]. This phlorotannin containing ten or more hydroxyl groups shows a relatively high antioxidant capacity.

Direct VEGF treatment and microgravity stimulation induced EMT, but IPA treatment reduced cell migration and EMT marker expression (Figure 5 and Figure 6). Therefore, IPA can be a therapeutic VEGFR2 inhibitor candidate for various angiogenesis-related eye diseases.

## 5. Conclusions

Actin cytoskeleton regulators were modulated in microgravity-stimulated ARPE19 cells. Excessive VEGF production and EMT marker expression also increased. However, IPA restored VEGF production and EMT by inhibiting VEGF–VEGFR2 binding. Therefore, IPA treatment effectively reduced VEGF-induced cell migration.

## Data Availability

Data is contained within the article.

## Supplementary Materials

The following supporting information can be downloaded at: https://www.mdpi.com/article/10.3390/antiox11112212/s1; Table S1: Primer list for quantitative real-time PCR; Table S2: Antibodies information for ELISA and Western blot; Figure S1: Cell viability under simulated microgravity on day 7; Figure S2: Computational docking prediction of benzimidazole urea–VEGFR2.
