# Supplementary material for: Ishophloroglucin A Ameliorates VEGF-Induced Epithelial-Mesenchymal Transition via VEGFR2 Pathway Inhibition in Microgravity-Stimulated Human Retinal Pigment Epithelial Cells
Source: Antioxidants (Basel). 2022 Nov 8;11(11):2212. doi: 10.3390/antiox11112212 (PMC9686763; doi:10.3390/antiox11112212)
Supplement: Supplementary file 1 [file antioxidants-11-02212-s001.zip › antioxidants-2016530-supplementary.pdf]

Supplementary Materials

Table S1. Primer list for quantitative real-time PCR.

Gene		Primers
ACTA2	Forward	5'-AAA AGA CAG CTA CGT GGG TGA-3′
	Reverse	5'-GCC ATG TTC TAT CGG GTA CTT C-3′
CDC42	Forward	5'-CCA TCG GAA TAT GTA CCG ACT G-3′
	Reverse	5'-CTC AGC GGT CGT AAT CTG TCA-3′
CDH2	Forward	5'-CCT CCA GAG TTT ACT GCC ATG AC-3′
	Reverse	5'-GTA GGA TCT CCG CCA CTG ATT C-3′
COLA1	Forward	5'-GTG CGA TGA CGT GAT CTG TGA-3′
	Reverse	5'-CGG TGG TTT CTT GGT CGG T-3′
FN1	Forward	5'-ACA ACA CCG AGG TGA CTG AGA C-3′
	Reverse	5'-GGA CAC AAC GAT GCT TCC TGA G-3′
MMP2	Forward	5'-CCC ACT GCG GTT TTC TCG AAT-3′
	Reverse	5'-CAA AGG GGT ATC CAT CGC CAT-3′
RAC1	Forward	5'-AAC CTG CCT GCT CAT CAG TT-3′
	Reverse	5'-TGA CAG CAC CGA TCT CTT T-3′
RHOA	Forward	5'-GGA AAG CAG GTA GAG TTG GCT-3′
	Reverse	5'-GGC TGT CGA TGG AAA AAC ACA T-3′
SNAI2	Forward	5'-CGA ACT GGA CAC ACA TAC AGT G-3′
	Reverse	5'-CTG AGG ATC TCT GGT TGT GGT-3′
VIM	Forward	5'-AGT CCA CTG AGT ACC GGA GAC-3′
	Reverse	5'-CAT TTC ACG CAT CTG GCG TTC-3′
GAPDH	Forward	5'-ACA AAG CTG TTC AGT GTC TCC A-3′
	Reverse	5'-CTC CGT TTC CAG AAT ACA CAC A-3′

Table S2. Antibody information for ELISA and Western blot.

Name	Company	Cat. number	Dilution rate	
			ELISA	Western blot
VEGF	Santa Cruz Biotechnology	sc-57496	1:200	-
TGFβ2	Santa Cruz Biotechnology	sc-374658	1:100	-
CTGF	Santa Cruz Biotechnology	sc-373936	1:100	-
VEGFR2	Bioss	bs-10412R	-	1:500
pErk1/2	Bioss	bsm-54491R	-	1:500
Erk1/2	Bioss	bsm-52259R	-	1:500
pAKT1/2/3	Santa Cruz Biotechnology	sc-271966	-	1:500
AKT1/2/3	Santa Cruz Biotechnology	sc-81434	-	1:500
α-SMA	Santa Cruz Biotechnology	sc-53142		1:500
Vimentin	Santa Cruz Biotechnology	sc-6260		1:500
GAPDH	Sigma-Aldrich	G8795	-	1:1,000

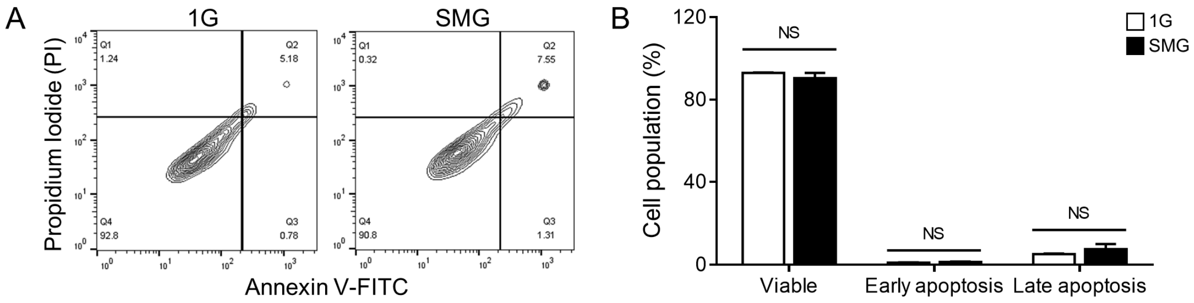

Figure S1. Cell viability under simulated microgravity on day 7. (**A**) Representative flow cytometry results using PI/Annexin V-FITC for apoptosis. ARPE19 cells were stimulated microgravity (SMG) for 7 days and the cells were stained with PI/Annexin V-FITC for flow cytometric analysis. (**B**) The cell population (%) of viable cells (Q4), early apoptotic cells (Q3) and late apoptotic cells (Q2) were statistically compared to 1G. FITC, fluorescein isothiocyanate; NS, Not Significant; PI, propidium iodide.

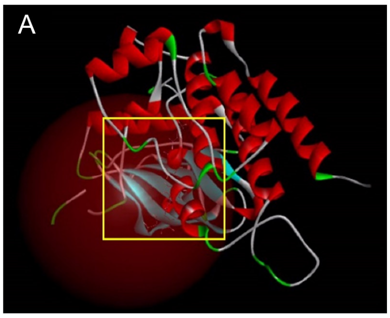

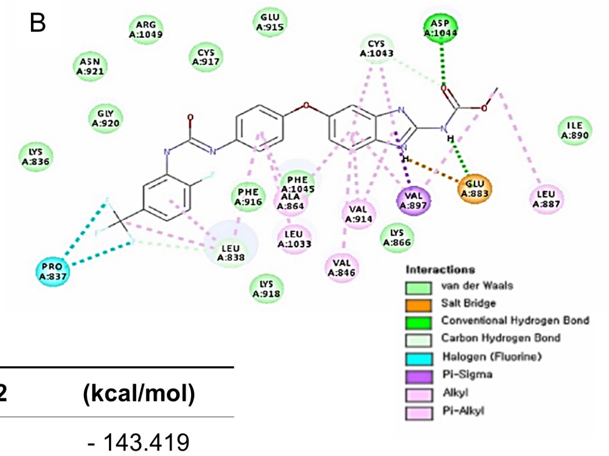

C

Benzimidazole urea-VEGFR2	(kcal/mol)
Binding energy	- 143.419
CDOCKER Interaction energy	- 57.0972

Figure S2. Computational docking prediction of the Benzimidazole urea-VEGFR2. (**A**) 3D diagram and (**B**) 2D diagram of Benzimidazole urea-VEGFR2 (marked in a yellow square) show the docking site. (**C**) The table indicates the results of the binding energy and CDOCKER interaction energy of the docking of Benzimidazole urea-VEGFR2. VEGFR2; Vascular endothelial growth factor receptor 2.

Supplementary Methods

Flow cytometry using PI/Annexin V-FITC staining for apoptosis analysis

ARPE19 cell apoptosis after microgravity stimulation was evaluated using an apoptosis detection kit (TransDetect®, Beijing, China) according to the manufacturer's methods. Microgravity stimulated (SMG) or normal gravity exposed (1G) ARPE19 cells were stained with 5 μL Annexin V-FITC and 5 μL PI at room temperature for 15 min in the dark. Stained ARPE19 cells were detected by flow cytometer (FACSCalibur; Beckman Coulter, IN, USA). A minimum of 10,000 cells were used for each analysis and the experiment was performed in triplicate.
